# Larger patients shouldn’t have fewer options: urethroplasty is safe in the obese

**DOI:** 10.1590/S1677-5538.IBJU.2019.0511

**Published:** 2020-09-02

**Authors:** Jordan Alger, Henry Collier Wright, Sameer Desale, Krishnan Venkatesan

**Affiliations:** 1 Department of Urology MedStar Georgetown University Hospital WashingtonDC USA Department of Urology, MedStar Georgetown University Hospital, Washington, DC, USA;; 2 MedStar Health Research Institute HyattsvilleMD USA MedStar Health Research Institute, Hyattsville, MD, USA;; 3 Department of Urology MedStar Washington Hospital Center WashingtonDC USA Department of Urology, MedStar Washington Hospital Center, Washington, DC, USA

**Keywords:** Urethral Stricture, Obesity, Recurrence

## Abstract

**Objective:**

To examine the impact of obesity on perioperative outcomes and urethral stricture recurrence after anterior urethroplasty.

**Material and Methods:**

We reviewed our prospectively maintained single-surgeon database to identify men with anterior urethral strictures who had undergone anastomotic or augmentation urethroplasty between October 2012 and March 2018. In all, 210 patients were included for primary analysis of perioperative outcomes, while 193 patients with at least 12 months follow-up were included for secondary analysis of stricture recurrence. Patients grouped by BMI were compared using univariate and multivariate analyses for perioperative outcomes and log rank testing for recurrence-free survival.

**Results:**

Overall, 41% (n=86) of patients were obese and 58.6% (n=123) had bulbar urethral strictures. Obese patients had significantly longer urethral strictures (mean=6.7cm±4.7) than nonobese patients (p <0.001). Though urethroplasty in obese patients was associated with increased estimated blood loss (EBL) relative to normal BMI patients on both univariate (p=0.003) and multivariate (p <0.001) analyses, there was no difference in operative time, length of stay, or complication rate between BMI groups. At a mean follow-up interval of 36.7 months, 15% (n=29) of patients had stricture recurrence, yet recurrence-free survival was not significantly different between groups (log rank p=0.299). Dorsal augmentation urethroplasty resulted in significantly fewer recurrences in obese patients compared to nonobese patients (p=0.036).

**Conclusion:**

Despite the association with increased urethral stricture length and EBL, obesity is not predictive of adverse perioperative outcomes or stricture recurrence. Obese patients should be offered urethral reconstruction, but patient selection and preoperative counseling remain imperative.

## INTRODUCTION

Urethral stricture disease can be burdensome for patients, both medically and socially. Urethroplasty is the gold standard for treatment and has success rates as high as 85-90% ( 1 ). However, these repairs are technically challenging and, risk of recurrence aside, are subject to complications including bleeding, infection, deep vein thrombosis, urethrocutaneous fistula, and erectile dysfunction.

Comorbidities conditions have been demonstrated as independent risk factors for complications after numerous surgical procedures ( 2 ). Among these, obesity is of particular interest given its worldwide prevalence --approximately 13% among adults worldwide based on a 2016 World Health Organization (WHO) estimate ( 3 ) -- and established association with other perioperative complications such as delayed wound healing and surgical site infections ( 4 , 5 ).

Recently, there has been uncertainty regarding the impact of obesity on surgical outcomes in patients with urethral stricture disease. Obesity has been associated with higher complication rates and urethroplasty failure ( 6 ), but it is not clear whether all perioperative measures are adversely affected in obese patients. Our aim was to examine the relationship between obesity, perioperative outcomes, and stricture recurrence after urethroplasty to ensure our patient selection has been appropriate and our counseling accurate. Based on existing data regarding obesity and perioperative complications, we hypothesized that obese patients have worse outcomes across a number of perioperative indexes including estimated blood loss, operative time, length of stay, and risk of complications. We also hypothesized that obese patients are prone to higher rate of urethral stricture recurrence.

## MATERIALS AND METHODS

### Patient selection and surgical technique

We reviewed our prospectively maintained, IRB-approved database of patients undergoing urethral stricture management at MedStar Washington Hospital Center between October 2012 and March 2018. All procedures were performed by a single fellowship-trained reconstructive urologist. Though consideration was given to stricture location, etiology, and tissue suitability, surgical technique selection was based primarily on stricture length as follows: i) excision and primary anastomosis (EPA) for <2cm ii) augmented anastomotic or augmentation urethroplasty for 2-5cm and iii) augmentation urethroplasty for >5cm. A preoperative retrograde urethrogram was performed to characterize stricture length and location. Perioperative parameters were obtained on the day of surgery, including patient height, weight, BMI, EBL, operative time, and stricture length, which was assessed intraoperatively using cystoscopy and ruler measurement. Stricture etiology was determined by patient history, and the diagnosis of lichen sclerosus (LS) was based on clinical exam or biopsy if necessary.

Our primary analysis included male patients who underwent either anastomotic or augmentation urethroplasty for anterior urethral stricture with at least three months follow-up after surgery. Penile, bulbar, and panurethral strictures were eligible. Both single and staged procedures were also eligible as were patients undergoing re-do urethroplasty after a failed initial urethroplasty. Posterior urethral stenosis is a pathologic entity that is distinctly different from anterior urethral stricture and is comparatively rare in our practice. Consequently, posterior urethroplasties were excluded due to insufficient statistical power and to prevent comparing non-equivalent conditions. Cases of isolated meatal stenosis, female urethroplasty, and urethroplasty for neophallus in transgender patients were also excluded.

### Follow-up

All patients were discharged home from surgery with an indwelling urethral catheter and oral prophylactic antibiotics. Patients underwent peri-catheter retrograde urethrogram at either two weeks postoperatively (for anastomotic urethroplasty) or three week postoperatively (for augmentation urethroplasty), and if no extravasation was seen the catheter was removed. All patients appeared for an additional follow-up appointment at 4 months and 12 months after surgery, and annually thereafter where an updated history and physical exam, uroflowmetry, and a post-void residual (PVR) were obtained. If at any time postoperatively there was concern for stricture recurrence based on symptomatology, uroflowmetry, or an elevated PVR, the patient underwent a retrograde urethrogram. For the determination of recurrence-free survival (i.e. urethral patency) in our secondary analysis, patients were required to follow-up at least 12 months after surgery.

### Outcomes

Primary outcomes for our study included estimated blood loss (EBL), operative time, hospital length of stay (LOS), and Clavien-Dindo ≥Grade III perioperative complications (i.e. requiring invasive surgical, endoscopic, or radiologic intervention) within 30 days of surgery. The principal secondary outcome was urethroplasty success at 12 months after surgery. Patients were further evaluated if they had symptoms of stricture recurrence (e.g. slowing urine stream), an elevated PVR, or a Qmax <10mL/s on uroflowmetry. Success was defined as having no need for further intervention (e.g. no cystoscopic dilation, urethrotomy, or self-calibration).

### Statistical analysis

All relevant patient data including age, Body Mass Index (BMI), stricture length, stricture location, and stricture etiology were obtained via review of the electronic medical records (EMR) and were de-identified within the database. Patients were stratified by BMI based on the following WHO definitions: normal weight (18.5 to <25kg/m^2^), overweight (25 to <30kg/m^2^), and obese (≥30kg/m^2^). Comparisons between patient characteristics within BMI groupings were made using one-way analysis of variance (ANOVA) for parametric continuous variables, a Kruskal-Wallis test for non-parametric continuous variables, and a Chi-square test for categorical variables. Univariate regression models were generated to assess the impact of BMI on primary and secondary outcomes. Multivariate regression analyses were also performed using the following variables: operative time, BMI, stricture location, and stricture etiology. To determine stricture recurrence-free survival, Kaplan-Meier curves were calculated and were compared using log rank testing.

A significance level of α=0.05 was used for all analyses and included calculation of 95% confidence intervals. All analyses were performed using a publically available statistical software package (R: R Foundation for Statistical Computing, Vienna, Austria).

## RESULTS

A total of 210 patients met the inclusion criteria for primary analysis of perioperative outcomes. Demographic statistics are displayed in Table-1 . Overall, 19% of patients had normal BMI (n=40), 40% were overweight (n=84), and 41% were classified as obese (n=86). Within this last group, 20 patients (9.5%) were morbidly obese with BMI ≥40kg/m2. Mean BMI was 30.16kg/m2 (SD±6.84). Mean patient age was 51 years old (±15.8) at the time of surgery and did not differ between BMI groups (p=0.202). The majority of strictures (58.6%, n=123) involved the bulbar urethra and 43.3% (n=91) of all strictures were idiopathic. There were no significant differences in stricture location between groups, however, etiologies varied with obese patients being more likely to have LS or an iatrogenic stricture than nonobese patients and less likely to have a history of genital trauma (p=0.036). The overall Clavien-Dindo ≥Grade III complication rate was 4.8% (n=10) and stricture recurrence was seen in 15.0% (n=29) of the 193 patients with at least 12 months follow-up data. In three cases (1.4%), patients developed metachronous urethral strictures away from the original site of urethral repair.

**Table 1:** Patient characteristics by BMI classification.

Variables		BMI Classification			
	
	All	Normal (BMI < 25)	Overweight (25 ≤ BMI < 30)	Obese (BMI ≥ 30)	P-value

	n=210	n=40 (19%)	n=84 (40%)	n=86 (41%)	
Age at Operation (mean ± SD)	51 ± 16	46 ± 19	51 ± 16	53 ± 14	0.202
Length (cm) of Stricture	4.2 ( 3 , 6 )	4 ( 2 , 5 )	4 ( 2 , 6 )	5 (4, 6.9)	**<0.0001**
(median, IQR)/ (mean ± SD)	5.7 ± 4.4	4.6 ± 3.5	5.1 ± 4.2	6.7 ± 4.7	
Weight (kg) (mean ± SD)	94.7 ± 25.6	70.0 ± 9.1	83.7 ± 12.6	116.8 ± 22.6	**<0.0001**
**Location of Stricture**					
Bulbar	123 (58.6%)	26 (65.0%)	51 (60.7%)	46 (53.5%)	0.508
Panurethral	30 (14.3%)	4 (10.0%)	12 (14.3%)	14 (16.3%)	
Penile	47 (22.4%)	10 (25.0%)	18 (21.4%)	19 (22.1%)	
Peno-Bulbar	10 (4.8%)	0 (0.0%)	3 (3.6%)	7 (8.1%)	
**Etiology**					
Hypospadias	14 (6.7%)	5 (12.5)	3 (3.6%)	6 (7%)	**0.036**
Iatrogenic	49 (23.3%)	4 (10%)	23 (27.4%)	22 (25.6%)	
Idiopathic	91 (43.3%)	17 (42.5%)	36 (42.9%)	38 (44.2%)	
Lichen Sclerosus	18 (8.6%)	1 (2.5%)	6 (7.1%)	11 (12.8%)	
Trauma	27 (12.9%)	10 (25%)	12 (14.3%)	5 (5.8%)	
Other	11 (5.2%)	3 (7.5%)	4 (4.8%)	4 (4.7%)	
**Technique**					
Dorsal Augmentation	102 (48.6%)	13 (32.5%)	38 (45.2%)	51 (59.3%)	**0.002**
EPA	37 (17.6%)	10 (25%)	22 (26.2%)	5 (5.8%)	
Perineal Urethrostomy	13 (6.2%)	2 (5%)	4 (4.8%)	7 (8.1%)	
Ventral @Augmentation	44 (21%)	9 (22.5%)	14 (16.7%)	21 (24.4%)	
Other	14 (6.7%)	6 (15%)	6 (7.1%)	2 (2.3%)	

At the time of surgery, obesity was associated with increased mean urethral stricture length, with obese patients having longer strictures (6.7cm±4.7) compared to normal BMI (4.6cm±3.5) and overweight (5.1cm±4.2) patients (p <0.001). As shown in Table-2 , there was also a BMI-associated increase in EBL from normal BMI to obese patients (198cc vs. 225cc vs. 270cc), but only the difference between normal BMI and obese patients was significant (p=0.028). Despite the associations of obesity with both stricture length and EBL, operative time, length of stay, and complication rate were not statistically different between BMI groups.

**Table 2:** Patient outcomes by BMI classification.

Variables		BMI Classification			
	All	Normal (BMI < 25)	Overweight (25 ≤ BMI < 30)	Obese (BMI ≥ 30)	P-value

	n=210	n=40 (19%)	n=84 (40%)	n=86 (41%)	
Length of Stay (days) (mean ± SD)	1.28 +1.08	1.27 + 0.88	1.32 + 1.45	1.25 + 0.69	0.903
Operative Time (min)	180 (150, 229)	150 (133, 231)	160 (150, 220)	180 (150, 235)	0.570
(median, IQR)/(mean ± SD)	188 ± 76	186 ± 84	184 ± 69	194 ± 80	
EBL (mL)	250 (150, 300)	200 (150, 250)	250 (138, 300)	250 (200, 300)	**0.028**
(median, IQR)/(mean ± SD)	238 ± 131	198 ± 82	225 ± 110	270 ± 158	
**Recurrence**					
No	164 (85.0%)	31 (83.8%)	63 (80.8%)	70 (89.7%)	0.285
Yes	29 (15.0%)	6 (16.2%)	15 (19.2%)	8 (10.3%)	
**Complications (Clavien-Dindo Grade ≥ III)**					
No	200 (95.2%)	40 (100%)	79 (94.0%)	81 (94.2%)	0.305
Yes	10 (4.8%)	0 (0%)	5 (6.0%)	5 (5.8%)	

In all groups, the majority of procedures performed were augmentation urethroplasties (69.5% overall), yet the distribution of techniques utilized differed significantly by BMI (p=0.002). Obese patients were more likely to undergo augmentation urethroplasty than normal weight patients (total 83.7% vs. 55%, respectively) and less likely to undergo excision with primary anastomosis (5.8% vs. 25%, respectively). Despite these differences, the only significant association between technique and either complication or recurrence rate was among obese patients undergoing dorsal augmentation, who were less likely to experience stricture recurrence than nonobese patients undergoing dorsal augmentation (4.2% vs. 20%, p=0.036; see Tables 3A and 3B ). Among “other” repairs performed, three were repeat procedures after a prior failed repair, four were part of a staged repair, and seven involved urethral reconstruction in the absence of stricture (e.g. repair of hypospadias or urethrocutaneous fistula).

**Table 3A:** Complications by BMI classification and technique.

		BMI Classification			
Technique	All	Normal (BMI < 25)	Overweight (25 ≤ BMI < 30)	Obese (BMI ≥ 30)	P-value

	n=210	n=40 (19%)	n=84 (40%)	n=86 (41%)	
Dorsal Augmentation	7/102 (6.7%)	0/13 (0.0%)	2/38 (5.3%)	5/51 (9.8%)	0.6385
EPA	0/37 (0.0%))	0/10 (0.0%)	0/22 (0.0%)	0/5 (0.0%)	-
Perineal Urethrostomy	188 ± 76	186 ± 84	184 ± 69	194 ± 80	0.4615
Ventral Augmentation	250 (150, 300)	200 (150, 250)	250 (138, 300)	250 (200, 300)	0.5227
Other	238 ± 131	198 ± 82	225 ± 110	270 ± 158	1.000

**Table 3B:** Recurrence by BMI classification and technique.

		BMI Classification			
Technique	All	Normal (BMI < 25)	Overweight (25 ≤ BMI < 30)	Obese (BMI ≥ 30)	P-value

	n=193	n=37 (19.2%)	n=78 (40.4%)	n=78 (40.4%)	
Dorsal Augmentation	12/98 (12.2%)	2/13 (15.4%)	8/37 (21.6%)	2/48 (4.2%)	**0.0362**
EPA	3/29 (10.3%)	1/8 (12.5%)	2/17 (11.8%)	0/4 (0.0%)	1.000
Perineal Urethrostomy	2/12 (16.7%)	1/2 (50.0%)	1/4 (25.0%)	0/6 (0.0%)	0.2273
Ventral Augmentation	10/40 (25.0%)	2/8 (25.0%)	3/14 (21.4%)	5/18 (27.8%)	1.000
Other	2/14 (14.3%)	0/6 (0.0%)	1/6 (16.7%)	1/2 (50.0%)	0.2747

To further assess the impact of BMI on outcomes, univariate and multivariate regression models were calculated. On univariate analysis (UVA), EBL --the only outcome associated with BMI classification-- was positively correlated with obesity, increased stricture length, and operative time. The presence of a penile stricture, history of hypospadias, and performance of either a perineal urethrostomy or “other” urethral procedure were predictive of lower EBL. There was no association between EBL and the presence of LS (p=0.584). For the multivariate analysis (MVA), age was excluded as it was not a significant predictor of EBL. Stricture length and surgical technique were found to be strongly correlated with location of stricture (p<0001) and hence were also excluded from the MVA to avoid multicollinearity. On MVA, obese patients experienced significantly greater EBL (p <0.0001) than normal BMI patients after controlling for operative time, stricture location, and stricture etiology. However, differences in EBL did not translate to significant differences in blood transfusion rates as only one patient in our entire cohort received a transfusion.

**Figure 1:**
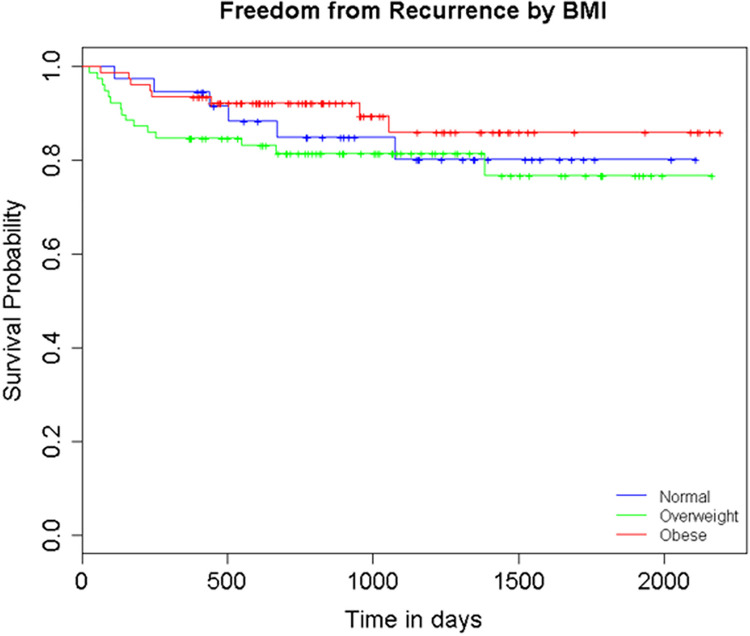
Recurrence by BMI (n=193)

Lastly, for our secondary outcome of stricture recurrence, Kaplan-Meier recurrence-free survival curves were calculated for the subset of 193 patients with at least 12 months of follow-up data ( Figure 1 ). The mean duration of follow-up was 36.7 months (SD±17.0) and overall there was a 15% stricture recurrence rate (n=29). We observed that recurrence rates between normal (16.2%), overweight (19%), and obese (10.3%) patients were not significantly different (p=0.285) and that there was no significant difference between survival curves (log rank p=0.299).

## DISCUSSION

The estimated incidence of urethral stricture in the United States ranges from 0.2-0.6%, resulting in annual treatment costs of around $200 million ( 7 , 8 ). In light of this volume, increasing attention has focused on improving treatment algorithms, with urethroplasty emerging as the gold standard for longer, more complex, or recurrent strictures ( 9 ). However, urethroplasty can be technically challenging with complication rates as high as 13-33% ( 10 - 12 ). Certain comorbidities have been linked to higher complication rates after other surgeries, yet studies assessing the impact of obesity on perioperative complications and stricture recurrence after urethroplasty have produced mixed results ( 13 - 17 ). Based on prior data and our own biases, we hypothesized that obesity, by virtue of the anatomy (e.g. deeper perineum, more intervening fat, added time obtaining adequate exposure, etc.), would lend increased technical difficulty to urethroplasty and result in higher rates of complication and recurrence.

Our institution has a robust referral base with a large proportion of obese patients (41% in the study cohort), which we felt warranted an examination of our operative experience. To our knowledge, this study represents the largest single-center, single-surgeon cohort of obese patients who have undergone urethroplasty in the literature to date. By comparison, a recent review by Blaschko et al. analyzing national trends among nearly 13.700 urethroplasties performed in the United States between 2000-2010 showed only 6.7% of patients were classified as obese ( 18 ). In another study, one of the largest series to assess the impact of obesity on recurrence after urethroplasty, only 11.2% of patients were obese ( 19 ). It is unclear why such a discrepancy in the reported proportion of obese patients undergoing urethroplasty exists, but perhaps differences in referral patterns and regional rates of obesity are contributing factors. It is also possible that surgeon selection bias in offering or withholding urethroplasty to obese patients plays a role.

In this study, it is important to note that obese patients had significantly longer mean urethral stricture length than nonobese patients. This is not a novel finding and may be due to relatively impaired vasculature, a chronic inflammatory milieu, and/or impaired wound healing characteristic of obese patients ( 4 ). Low testosterone, which has been linked to urethral stricture disease, may also play a role given that obese males are more susceptible to hypogonadism ( 20 , 21 ). Alternatively, obese patients may simply experience delays in obtaining definitive care or be offered minimally invasive therapies (e.g. dilations) more readily than nonobese patients, leading to more extensive spongiofibrosis, as others have suggested ( 6 ).

Regardless of the reason, the differences in stricture length between obese and nonobese patients appeared to be of minimal clinical significance since outcomes, aside from EBL, did not differ in our study. Obese patients, for instance, did not endure a significantly higher rate of Clavien-Dindo Grade ≥ III complications than nonobese patients, though we acknowledge that only overweight and obese patients in this study experienced complications. Intraoperatively, we observed that obese patients did not uniformly have increased adipose deposition in the perineum as we had anticipated, suggesting that the urethral dissection may not necessarily have been more difficult or complicated. To be sure, EBL was significantly greater in obese patients after controlling for stricture length, but the clinical impact of this difference was not readily discernible as both blood transfusion rates and hospital LOS were similar to those of nonobese patients. Moreover, despite the increased prevalence of LS in obese patients, LS was not associated with EBL or LOS on MVA (p=0.58 and p=0.34, respectively).

A corollary of having had longer strictures is that obese patients were more likely to undergo augmentation urethroplasty and less likely to undergo EPA. Interestingly, dorsal augmentation as a technique was not associated with greater EBL relative to EPA on univariate analysis, and it was associated with a lower risk of recurrence in obese patients relative to nonobese patients. No other technique, including PU, in any BMI group had an association with risk of recurrence. Consequently, we believe that the full breadth of urethral reconstructive techniques can be employed successfully in obese patients and that BMI should not be used as a parameter for selecting surgical approach.

With respect to urethroplasty success, patients overall in our study had similar recurrence rates to those previously reported, regardless of BMI. Though this seems to contradict prior literature, the relationship between BMI and stricture recurrence may not be straightforward. Breyer et al., for example, found that obesity predicted stricture recurrence, yet patients with class II and class III obesity (BMI 35-40kg/m2 and BMI ≥40kg/m2, respectively) did not have a higher risk of recurrence after urethroplasty than class I obese patients (BMI 30-35kg/m2) ( 13 ). Similarly, on subset analysis of 20 morbidly obese patients in our study, we were surprised to find an equivalent complication rate (5%) and that, of the 17 patients with at least 12 months follow-up, no patients had recurred. Taken together, these findings suggest the effect of obesity on recurrence is not purely linear and may be influenced by other factors.

A problem in the literature is that a standardized definition of “success” after urethroplasty is not well established. We used patient subjective measures (relevant symptoms) as well as objective measures (uroflowmetry, PVR) to screen for stricture recurrence. However, other objective measures such as urethrography or cystoscopy, though more invasive and less accepted by patients, are likely to be more accurate for assessing recurrence. Though we did not standardize the subjective measures, validated questionnaires such as the International Prostate Symptom Score (IPSS) have been shown to lack accuracy in detecting stricture recurrence ( 22 ), and more sophisticated patient reported outcome measures (PROMs) are not yet standard use.

There are several limitations to this study. Firstly, this is a retrospective review of a single-surgeon experience, which could invoke selection bias and limits generalizability. An attempt to evaluate the relationship between BMI and outcomes after urethroplasty using a multi-center, prospective design must be undertaken. Secondly, a power analysis was not performed as part of the initial study design, which may have affected the ability to detect small differences in outcomes. However, our cohort size and rates of both complication and recurrence were similar to those of other published studies, so we did not feel this was a major limitation. Thirdly, we did not tabulate comorbid conditions such as smoker status or diabetes that may independently predict certain outcomes. Fourthly, we included PU in the analysis to better characterize the breadth of surgical options available to obese patients with urethral stricture disease. However, PU was performed more often in obese patients and may be perceived as a technically simpler procedure with superior outcomes. To that end, we feel it is important to note that while EBL was indeed lower in patients undergoing PU, the rate of recurrence after PU was similar to that of all other techniques (16.7% vs. 14.9%, respectively) and that the only two instances of recurrence after PU occurred in non-obese patients. Lastly, using a minimum 12 month follow-up for evaluating stricture recurrence may have been insufficient. Nevertheless, there is evidence suggesting 75% of recurrences occur within the first 6 months after surgery ( 16 ). Barbagli et al. similarly showed that, at a median follow-up of 118 months, 96% of recurrences after substitution urethroplasty occurred within 5 years and that a majority (40%) occurred within the first year ( 17 ). Based on this data, we felt 12 months was a reasonable time point for retrospection, but it is possible that patients differentially recur beyond this window. Lichen sclerosus, for example, has previously been associated with higher rates of stricture recurrence, yet there was no such association within the first 12 months on a subset analysis in our study (p=1.00). This underscores why long-term follow-up is essential for patients after urethroplasty.

Despite the aforementioned limitations, the results of this study support the null hypothesis and demonstrate that anterior urethroplasty can be performed safely and successfully in obese patients. There was no increased risk of complications or recurrence in obese patients, suggesting there is no need to follow these patients more closely than nonobese patients. Though obese patients may not presently be offered anterior urethroplasty as consistently as non-obese patients, based on our experience they probably should be.

## CONCLUSIONS

Though associated with increased stricture lengths, obesity does not preclude successful urethral reconstruction. Blood loss may be higher in obese patients undergoing urethroplasty, but its clinical significance appears to be negligible. When considering surgical approach for an obese patient, it is worth considering that dorsal augmentation urethroplasty offers an equivalent or possibly lower risk of stricture recurrence. Overall, obese patients can and should be offered urethral reconstruction, but careful patient selection and thorough preoperative evaluation and counseling remain imperative.

## References

[1] 1. Hampson LA, McAninch JW, Breyer BN. Male urethral strictures and their management. Nat Rev Urol. 2014;11:43-50. 10.1038/nrurol.2013.275 PMC4123447 24346008

[2] 2. Breyer BN, McAninch JW, Whitson JM, Eisenberg ML, Mehdizadeh JF, Myers JB, et al. Multivariate analysis of risk factors for long-term urethroplasty outcome. J Urol. 2010;183:613-7. 10.1016/j.juro.2009.10.018 20018318

[3] 3. World Health Organization. Obesity and Overweight. World Health. Available at. <http://www.who.int/news-room/fact-sheets/detail/obesity-and-overweight> accessed Feb 16, 2018.

[4] 4. Pierpont YN, Dinh TP, Salas RE, Johnson EL, Wright TG, Robson MC, et al. Obesity and surgical wound healing: a current review. ISRN Obes. 2014;2014:638936. 10.1155/2014/638936 PMC3950544 24701367

[5] 5. Winfield RD, Reese S, Bochicchio K, Mazuski JE, Bochicchio GV. Obesity and the Risk for Surgical Site Infection in Abdominal Surgery. Am Surg. 2016;82:331-6. 27097626

[6] 6. Privratsky JR, Almassi N, Guralnick ML, Anderson BJ, O’Connor RC. Outcomes of grafted bulbar urethroplasty in men with class II or III obesity. Urology. 2011;78:1420-3. 10.1016/j.urology.2011.07.1429 22014595

[7] 7. Alwaal A, Blaschko SD, McAninch JW, Breyer BN. Epidemiology of urethral strictures. Transl Androl Urol. 2014;3:209-13. 10.3978/j.issn.2223-4683.2014.04.07 PMC4708169 26813256

[8] 8. Santucci RA, Joyce GF, Wise M. Male urethral stricture disease. J Urol. 2007;177:1667-74. 10.1016/j.juro.2007.01.041 17437780

[9] 9. American Urological Association. (2016). Male Urethral Stricture. Available at. <http://www.auanet.org/guidelines/urethral-stricture-(2016)>.

[10] 10. Meeks JJ, Erickson BA, Granieri MA, Gonzalez CM. Stricture recurrence after urethroplasty: a systematic review. J Urol. 2009;182:1266-70. 10.1016/j.juro.2009.06.027 19683309

[11] 11. Navai N, Erickson BA, Zhao LC, Okotie OT, Gonzalez CM. Complications following urethral reconstructive surgery: a six year experience. Int Braz J Urol. 2008;34:594-600; discussion 601. 10.1590/s1677-55382008000500008 18986563

[12] 12. Andrich DE, Dunglison N, Greenwell TJ, Mundy AR. The long-term results of urethroplasty. J Urol. 2003;170:90-2. 10.1097/01.ju.0000069820.81726.00 12796652

[13] 13. Breyer BN, McAninch JW, Whitson JM, Eisenberg ML, Master VA, Voelzke BB, et al. Effect of obesity on urethroplasty outcome. Urology. 2009;73:1352-5. 10.1016/j.urology.2008.12.073 PMC4113077 19371937

[14] 14. Gimbernat H, Arance I, Redondo C, Meilán E, Ramón de Fata F, Angulo JC. Analysis of the factors involved in the failure of urethroplasty in men. Actas Urol Esp. 2014;38:96-102. 10.1016/j.acuro.2013.07.003 24051326

[15] 15. Kessler TM, Schreiter F, Kralidis G, Heitz M, Olianas R, Fisch M. Long-term results of surgery for urethral stricture: a statistical analysis. J Urol. 2003;170:840-4. 10.1097/01.ju.0000080842.99332.94 12913712

[16] 16. Kinnaird AS, Levine MA, Ambati D, Zorn JD, Rourke KF. Stricture length and etiology as preoperative independent predictors of recurrence after urethroplasty: A multivariate analysis of 604 urethroplasties. Can Urol Assoc J. 2014;8:E296-300. 10.5489/cuaj.1661 PMC4039590 24940453

[17] 17. Barbagli G, Kulkarni SB, Fossati N, Larcher A, Sansalone S, Guazzoni G, et al. Long-term followup and deterioration rate of anterior substitution urethroplasty. J Urol. 2014;192:808-13. 10.1016/j.juro.2014.02.038 24533999

[18] 18. Blaschko SD, Harris CR, Zaid UB, Gaither T, Chu C, Alwaal A, et al. Trends, utilization, and immediate perioperative complications of urethroplasty in the United States: data from the national inpatient sample 2000-2010. Urology. 2015;85:1190-1194. 10.1016/j.urology.2015.01.008 PMC4917203 25746579

[19] 19. Chapman D, Kinnaird A, Rourke K. Independent Predictors of Stricture Recurrence Following Urethroplasty for Isolated Bulbar Urethral Strictures. J Urol. 2017;198:1107-1112. 10.1016/j.juro.2017.05.006 28483575

[20] 20. Hofer MD, Kapur P, Cordon BH, Hamoun F, Russell D, Scott JM, et al. Low Testosterone Levels Result in Decreased Periurethral Vascularity via an Androgen Receptor-mediated Process: Pilot Study in Urethral Stricture Tissue. Urology. 2017;105:175-80. 10.1016/j.urology.2017.02.037 28263822

[21] 21. Spencer J, Mahon J, Daugherty M, Chang-Kit L, Blakely S, McCullough A, et al. Hypoandrogenism is Prevalent in Males With Urethral Stricture Disease and is Associated with Longer Strictures. Urology. 2018;114:218-23. 10.1016/j.urology.2017.10.057 29378279

[22] 22. Tam CA, Elliott SP, Voelzke BB, Myers JB, Vanni AJ, Breyer BN, et al. The International Prostate Symptom Score (IPSS) Is an Inadequate Tool to Screen for Urethral Stricture Recurrence After Anterior Urethroplasty. Urology. 2016;95:197-201. 10.1016/j.urology.2016.04.006 PMC5002376 27109599

